# Cholera outbreak investigation in Adamawa state, Nigeria, June 2021 – December 2021: An unmatched case-control study

**DOI:** 10.4102/jphia.v17i1.1559

**Published:** 2026-08-20

**Authors:** Aisha A. Abba, Celestine Ameh, Aisha Sadauki, Muhammad S. Balogun, Muntari Hassan, Oladipo Ogunbode, Suleiman I. Ahmad, Lukman Ismaila, Peace Umar, Azuka Adeke

**Affiliations:** 1 Department of Health Emergency Preparedness and Response, Nigeria Centre for Disease Control and Prevention, Abuja, Nigeria; 2 Nigeria Field Epidemiology Training Programme, Abuja, Nigeria; 3 Africa Field Epidemiology Network, Abuja, Nigeria; 4 Department of Planning, Research and Statistics, Nigeria Centre for Disease Control and Prevention, Abuja, Nigeria; 5 Nigeria Centre for Disease Control and Prevention, Abuja, Nigeria; 6 Department of Surveillance and Epidemiology, Nigeria Centre for Disease Control and Prevention, Abuja, Nigeria

**Keywords:** cholera, outbreak, attack rate, case fatality rate, Nigeria

## Abstract

**Background:**

Cholera is a major public health challenge in Nigeria. On 09 September 2021, the Adamawa State Ministry of Health confirmed an outbreak affecting 12 local government areas (LGAs).

**Aim:**

To describe the outbreak and identify risk factors.

**Setting:**

Community-based in Adamawa State, Nigeria (30 June 2021 – 01 December 2021).

**Methods:**

We described the State cholera line list (30 June 2021 – 01 December 2021) and estimated case fatality and attack rates. We conducted an unmatched case-control study in seven LGAs, involving 66 laboratory-confirmed cases and 120 asymptomatic neighbourhood controls. We collected data on demographics, clinical history, practices and risk factors using interviewer-administered questionnaires. We executed univariate, bivariate and multivariate analyses to estimate adjusted odds ratios (aORs) and 95% confidence intervals (CIs).

**Results:**

Of 1332 cases reported across 12 LGAs, we recorded an overall case fatality rate (CFR) of 3.6%. We recorded the highest CFR (16.7%) in Song and the attack rate (*N* = 239.6/100 000) in Numan. Children aged 5–14 years were most affected. The median age was 24 years for cases (range: 1–76) and 32 years for controls (range: 5–70). Males accounted for 54.5% (cases) and 53.3% (controls). Independent risk factors were age < 10 years (aOR = 9.7; 95% CI: 2.6–34.9) and consumption of street-vended noodles (aOR = 7.0; 95% CI: 1.4–34.8).

**Conclusion:**

The outbreak disproportionately affected children with unregulated food sources, contributing significantly. Public health interventions included hygiene education and chlorine distribution. Strengthening food safety regulations and sustaining hygiene promotion were recommended to mitigate future outbreaks.

**Contribution:**

The study supports Nigeria’s Cholera Control Programme in refining hygiene strategies, regulating street food safety and targeting high-risk groups, such as young children.

## Introduction

Cholera persists as a major global health problem that causes substantial mortality and morbidity, particularly in low- and middle-income countries. The disease is caused by *Vibrio cholerae* and is primarily transmitted through contaminated food or water in settings with poor sanitation and unsafe drinking water.^1,2^ Despite global progress, recurrent outbreaks continue to occur, especially in Africa and Asia, the Middle East, and South and Central America, where weak health systems and humanitarian crises exacerbate transmission.^3,4^ In 2021, 23 countries reported cholera outbreaks, mostly within the African and Eastern Mediterranean Regions.^5^ This trend continued into 2022, with the number of affected countries increasing to 30.^5^ Africa continues to carry a large proportion of the global burden in comparison to other regions.^6^ African countries contributed 21% of the suspected global cholera cases and accounted for nearly 80% of the deaths reported to the World Health Organization (WHO) between 2014 and 2021.^7^ In 2022 alone, 15 African countries reported almost 80 000 cases and 1863 deaths.^8^

Nigeria is among the African countries where cholera is endemic, and the disease has been responsible for recurrent large-scale outbreaks.^8^ More than 43 000 cases and 836 deaths were reported across 20 states in 2018.^8^ A more recent outbreak occurred in 2021, when more than 111 000 suspected cases and 3600 deaths were documented nationwide, resulting in a case fatality rate (CFR) of 3.2%.^1,2^ Although the number of suspected cases had declined to approximately 24 000 with 592 deaths by 2022 (CFR of 2.5%), the outbreak remained geographically widespread, affecting 33 states.^9,10^ As in previous years, the northern part of the country, particularly the North-East, experienced the heaviest toll.^11^ Multiple factors contribute to the persistence of cholera in Nigeria, including unsafe water sources, overcrowded settlements and poor sanitation. In addition, humanitarian cases such as population displacement increase vulnerability to outbreaks by creating conditions that favour contamination of food and water supplies.^4,12,13^

Adamawa State, located in north-eastern Nigeria, was among the states affected during the 2021 cholera outbreak. Despite an extensive network of rivers and streams, sanitation remains a major public health challenge in the state, nearly 20% of the population practices open defecation, and access to safe drinking water and improved sanitation facilities is limited in many communities, creating conditions that facilitate recurrent outbreaks of waterborne diseases, including cholera.^14^ The Adamawa State Ministry of Health officially declared a cholera outbreak following reports of acute watery diarrheal illness across several local government areas (LGAs). The outbreak spread rapidly across multiple communities, resulting in substantial morbidity and mortality.^15^ In response, an incident management system was activated with partner support, and a cholera treatment centre was established in Yola to strengthen coordination and case management. However, while outbreak situation reports often emphasise case counts and transmission trends, there remains a paucity of evidence on the specific risk factors driving cholera transmission during outbreaks in Nigeria.^16^ Addressing this gap is essential for informing targeted prevention strategies and guiding timely public health action. We therefore investigated the 2021 cholera outbreak in Adamawa State to determine its magnitude, describe cases by person, place and time, and identify risk factors associated with the infection.

## Research methods and design

### Study design

We conducted a mixed-methods outbreak investigation comprising two components: (1) a descriptive epidemiological analysis of the Adamawa state of cholera cases reported in Adamawa State between 30 June 2021 and 01 December 2021, and (2) an unmatched case-control study conducted during the late phase of the outbreak to identify factors associated with cholera transmission.

The descriptive component utilised the Adamawa State cholera line list to characterise the outbreak by person, place and time. The analytic component was conducted between 19 November 2021 and 01 December 2021, corresponding to the period of active field investigation when laboratory confirmation capacity and incident case detection were optimal.

Seven LGAs (Demsa, Fufore, Girei, Mubi South, Numan, Yola North and Yola South) reporting new cases of cholera were included in the analytical study. These LGAs were purposively selected to capture areas with ongoing transmission, thereby enabling inclusion of recent incident cases and improving the accuracy of exposure assessment. Restricting the analytical study to LGAs with active case reporting minimised recall bias and enhanced the ability to identify current risk factors driving transmission.

### Setting

Adamawa State (capital Yola), located in the north-eastern part of Nigeria, has a population of 4 248 436 as of 2016.^17^ The state has 21 LGAs and is characterised by diverse climatic conditions, with annual rainfall ranging from 79 mm in the northern zone to 179 mm in the southern zone.^14^ Several rivers traverse the state, most notably the River Benue, which serves as a major source of water for domestic use, agriculture and livestock across multiple towns and communities.^17^

### Case definition for descriptive epidemiology

Case definitions for the descriptive component were adapted from standard cholera surveillance and outbreak investigation in the integrated disease surveillance and response technical guidelines issued by the Federal Ministry of Health, Nigeria Centre for Disease Control and Prevention, and the WHO.^18^ A suspected case of cholera was defined as any person residing in Adamawa State during the study period who presented with acute watery diarrhoea, with or without vomiting.^18^ An epidemiologically linked cholera case was defined as any suspected case with a documented link to a confirmed case by person, place or time. A confirmed case was defined as a suspected case with laboratory confirmation of *V. cholerae* O1 or O139 from a stool specimen or a positive rapid diagnostic test.^18^

### Case and control definitions for the analytic study

For the analytic component, a more specific case definition was employed to reduce outcome misclassification.

A case was defined as any person residing in Adamawa State with laboratory-confirmed *V. cholerae* infection whose illness onset occurred between 19 November 2021 and 01 December 2021.

A control was defined as any individual residing in the same neighbourhood as a confirmed case who reported no history of diarrhoea during the outbreak period.

Only laboratory-confirmed cases were included in the analytic study. Suspected and epidemiologically linked cases were used exclusively for descriptive epidemiology.

### Sampling strategy and participant selection for analytical study

Confirmed cases were identified from health facilities and cholera treatment centres across the seven selected LGAs.

Controls were selected from the same neighbourhoods as cases to enhance comparability of environmental and community-level exposures, such as water sources and sanitation conditions. Neighbourhoods were operationally defined as households within the immediate residential vicinity of a case household.

Control recruitment followed a systematic household sampling approach. Starting from the household of an identified case, field teams visited the second household to the right. In each selected household, all eligible household members were listed, and one individual was randomly selected as a control. Only one control was enrolled per household, even when multiple cases were identified. If a selected household declined participation or had no eligible participants, the next household was approached.

This study was unmatched; no individual matching by age, sex or other characteristics was performed.

### Eligibility criteria

Cases were eligible if they had laboratory-confirmed *V. cholerae* infection during the study period and resided in Adamawa State. Controls were eligible if they resided in the same neighbourhood as a confirmed case and reported no history of diarrhoea during the outbreak period.

Participants were excluded if they declined to provide informed consent or were unable to participate in the interview because of severe illness or other limiting conditions.

### Sample size

The sample size was determined using the StatCalc function in Epi Info version 7.2.4.0 (CDC, Atlanta, GA, United States [US]) based on a 95% two-sided confidence level, 80% power, and a case-to-control ratio of 1:2, with the Kelsey output option applied. An estimated 15.0% prevalence of exposure among controls derived from a previous outbreak informed the calculation.^19^ Based on these parameters, the minimum required sample size was 66 cases and 132 controls.

Given the evolving nature of the outbreak and field logistics, 120 controls were ultimately enrolled, resulting in a slightly reduced control-to-case ratio. All eligible laboratory-confirmed cases identified during the study period were included.

### Data collection

Data for the descriptive component were obtained from case line lists compiled by the State Ministry of Health, supplemented by active case searches conducted in health facilities and affected communities.

An interviewer-administered structured questionnaire developed and deployed using the Kobo toolbox (Kobo Inc., Cambridge, MA, US) was used to collect data from cases and controls for the analytical component. The questionnaire captured socio-demographic characteristics, clinical information, knowledge, attitude, and practices related to cholera, and potential exposure risk factors.

### Data analysis

#### Descriptive epidemiology

The dataset was systematically cleaned, and variables of interest included age, sex, date of symptom onset, LGA and outcome. Analysis was conducted by person, place and time. Frequencies, proportions, CFR, and attack rates were calculated using Microsoft Excel 2019 and Epi Info version 7.

#### Analytical epidemiology

The dataset was cleaned using Microsoft Excel 2019 and analysed with Epi Info version 7.2.4.0. Univariate, bivariate and multivariate analyses were performed. Key exposure variables, such as handwashing before meals, drinking water storage practices and age group, were dichotomised to facilitate bivariate analysis. Adjusted odds ratios with corresponding 95% confidence intervals (CIs) were calculated to measure associations between exposures and outcomes.

### Ethical considerations

Ethical clearance to conduct this study was obtained from the Adamawa State Health Research Ethics Committee (ADHREC), Nigeria (Protocol No. ADHREC 04/07/2025; Approval No. ADHREC 10/07/2025/37). The study received expedited ethical approval, and all research activities were conducted in accordance with the ethical principles and guidelines governing health research in Nigeria. Both cases and controls provided verbal consent for the interview.

## Results

### Descriptive epidemiology of the outbreak

**FIGURE 1:**
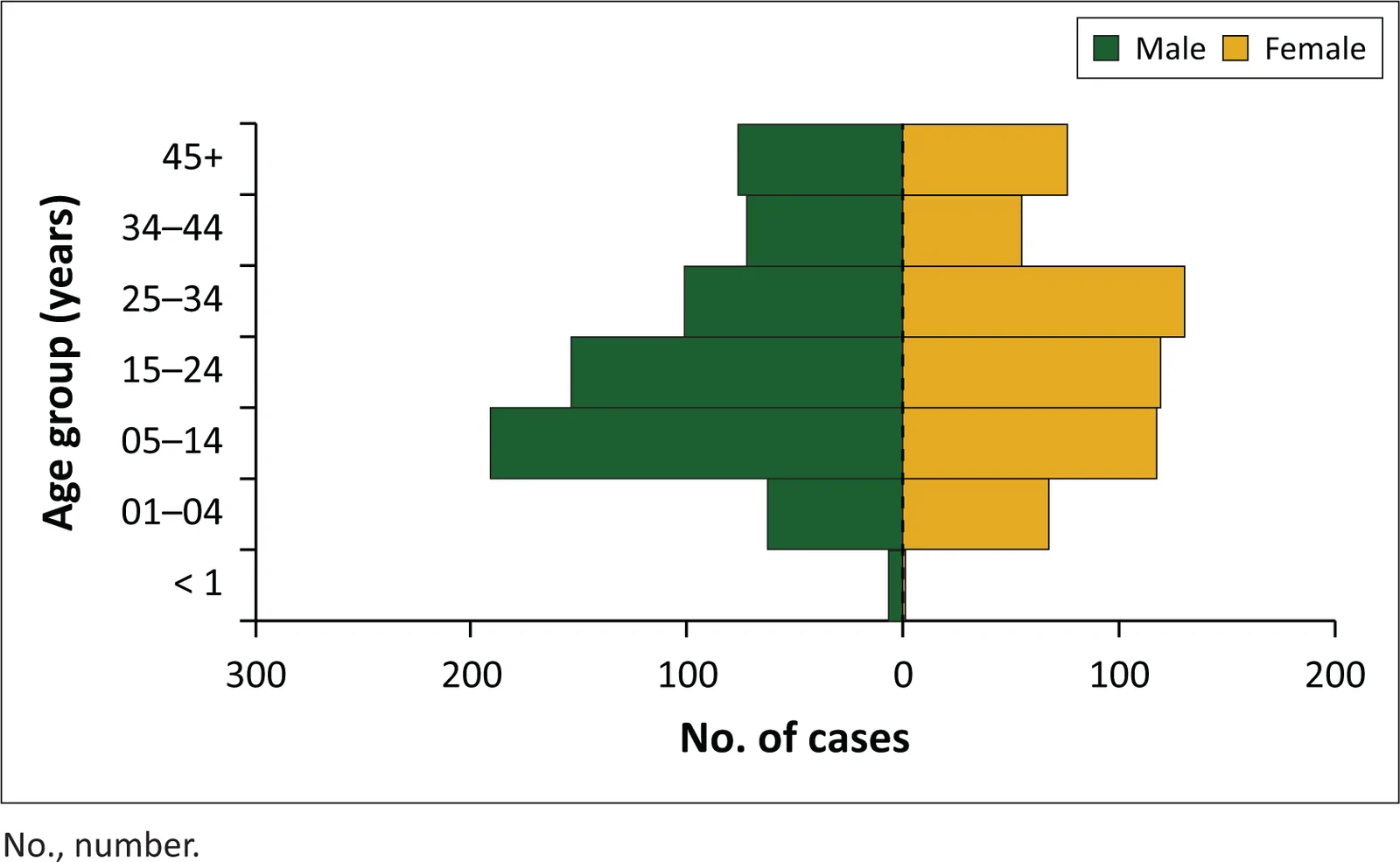
Age–sex distribution of cholera cases in Adamawa State, June 2021 to December 2021.

As of 22 December 2021, a total of 1332 cholera cases were reported across 12 LGAs in Adamawa State. The 5–14-year age group was the most affected, and more cases occurred among males than females (Figure 1). Yola North LGA accounted for the highest proportion of cases, 415 (31.2%), while Song LGA reported the lowest, 6 (0.2%). The overall CFR was 3.6%, with Song LGA recording the highest CFR (16.7%). The attack rate was highest in Numan LGA (*n* = 239.6 per 100 000 population) and lowest in Song LGA (*n* = 2.3 per 100 000 population) (Table 1).

**TABLE 1:** Demographic distribution of cholera cases in Adamawa State as of 22 December 2021.

LGA	Cases		Death	CFR (%)	LGA projected population	AR/100 000
	*n*	%				
Demsa	15	1.1	0	0	238 400	6.3
Fufore	29	2.2	0	0	279 900	10.4
Girei	150	11.3	3	2.0	173 500	86.5
Gombi	35	2.6	3	8.6	197 500	17.7
Hong	15	1.1	2	13.3	226 100	6.6
Lamurde	137	10.3	11	8.0	148 700	92.1
Mubi South	19	1.4	2	10.5	173 700	10.9
Numan	293	22.0	9	3.1	122 300	239.6
Shelleng	26	2.0	0	0	198 400	13.1
Song	6	0.5	1	16.7	260 900	2.3
Yola North	415	31.2	10	2.4	266 800	155.5
Yola South	192	14.4	7	3.6	262 200	73.2

**Total**	**1332**	**100**	**48**	**3.6**	**2 548 400**	**714.2**

Note: This data is based on an unpublished Cholera outbreak linelist, used as an internal document containing outbreak data from Adamawa State Ministry of Health, Adamawa State, Nigeria in December 2021.

AR, attack rate; CFR, case fatality rate; LGA, local governmental area.

**FIGURE 2:**
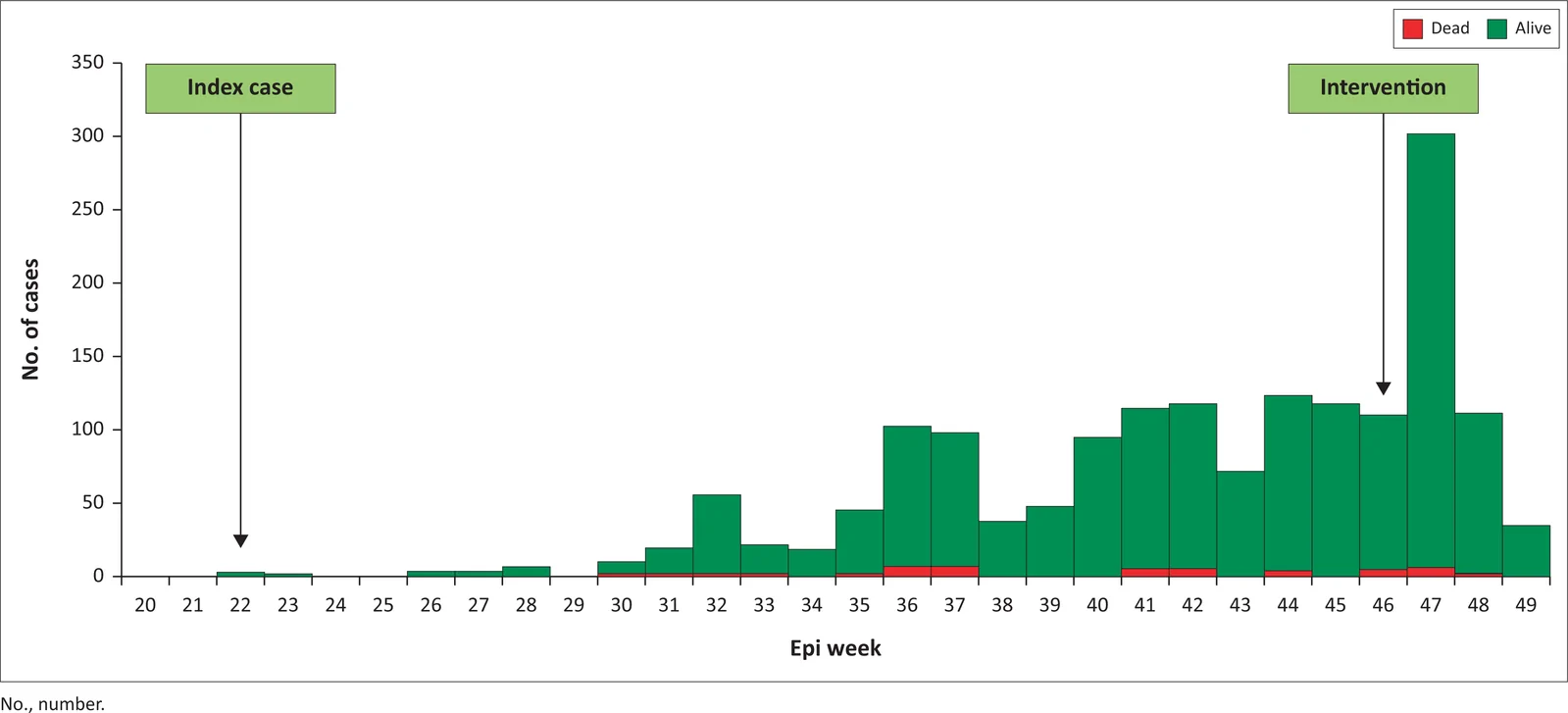
Epi curve by week of suspected cholera cases and deaths in Adamawa State from June 2021 to December 2021.

Figure 2 illustrates the epidemic curve of the outbreak. The index case was reported on 30 June 2021, in epidemiological week 22. Sporadic cases were identified from epidemiological week 26 to week 36, during which cases peaked, and deaths were reported. Cases steadily increased until week 46, when the rapid response team was dispatched to intervene.

**FIGURE 3:**
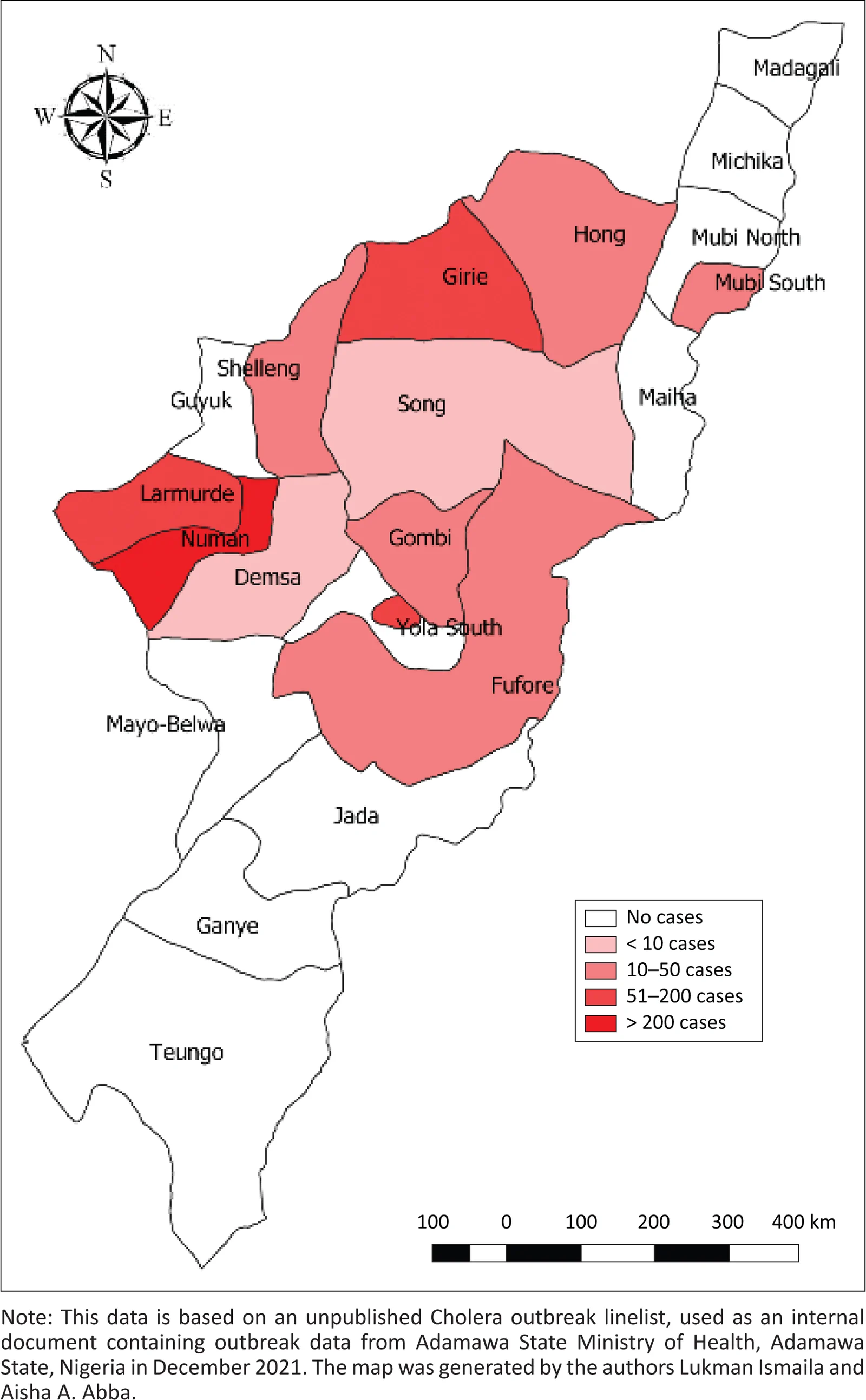
Map of Adamawa State showing local government areas affected by cholera.

Figure 3 shows that a higher number of cases (> 200) were seen in Yola North LGA, and the least number of cases (< 10) were seen in Song LGA.

### Analytical epidemiology

A total of 186 participants were enrolled in the case-control study, comprising 66 cases and 120 controls, yielding a 100% response rate. The median age across both groups was 28.0 years. Males represented 36 (54.5%) of the cases and 64 (53.3%) of the controls. Participants aged 30 years and above accounted for 22 (33.3%) of cases and 66 (55.0%) of controls. The majority of participants identified as Muslim, 56 (84.8%) among cases and 93 (77.5%) among controls. Unemployment was common among both groups, with 56 (84.8%) of cases and 99 (82.5%) of controls reporting no formal employment (Table 2).

**TABLE 2:** Socio-demographic distribution of **c**ases and **c**ontrols in Adamawa State, Nigeria, June 2021 – December 2021.

Characteristics	Cases		Controls		Total	
	*n*	%	*n*	%	*n*	%
**Sex**						
Male	36	54.5	64	53.3	100	53.7
Female	30	45.5	56	46.7	86	46.4
Total	66	35.5	120	64.5	186	100
**Age group (years)**						
0–9	13	19.7	3	2.5	16	8.6
10–19	13	19.7	21	17.5	34	18.3
20–29	18	27.3	30	25.0	48	25.8
≥ 30	22	33.3	66	55.0	88	47.3
**Religion**						
Islam	56	84.8	93	77.5	149	80.1
Christianity	10	15.2	27	22.5	37	19.9
**Type of household**						
Multi-dwelling	27	40.9	51	42.5	78	41.9
Single dwelling	39	59.1	69	57.5	108	58.1
**Occupation**						
Artisan	1	1.5	0	0.0	1	0.5
Banker	0	0.0	1	0.8	1	0.5
Business owner	2	3.0	5	4.2	7	3.8
Civil servant	3	4.5	13	10.8	16	8.6
Farmer	1	1.5	0	0.0	1	0.5
Others	3	4.5	2	1.7	5	2.7
Unemployed	56	84.8	99	82.5	155	83.3

Table 3 shows the risk factors associated with cholera infection amongst cases and controls. Those aged < 10 years are 9.7 times more likely to have cholera compared to those > 10 years (OR = 9.7, CI: 2.6–34.9). The table also shows that those who ate noodles from street vendors were seven times as likely to have cholera as those who did not (OR = 7.00, CI: 1.4–34.8).

**TABLE 3:** Multivariate analysis of selected exposures among cases and controls (*N* = 186).

Exposure	Cases (*N* = 66)		Controls (*N* = 120)		OR	OR (95% CI)	*p*-value	aOR	aOR (95% CI)
	*n*	%	*n*	%					
**Sex**	-	-	-	-	0.95	0.52–1.74	0.870	-	-
Male	36	54.5	64	53.3	-	-	-	-	-
Female	30	34.9	56	46.7	-	-	-	-	-
**Age group (years)**	-	-	-	-	9.56†	2.61–34.98†	< 0.001†	10.82†	2.94–9.72†
< 10	13	19.7	3	2.5	-	-	-	-	-
≥ 10	53	80.3	117	97.5	-	-	-	-	-
**Education**	-	-	-	-	1.31	0.63–2.81	0.257	-	-
Formal	44	66.7	73	60.8	-	-	-	-	-
Informal	27	18.2	26	21.6	-	-	-	-	-
**Household type**	-	-	-	-	0.93	0.50–1.72	0.830	-	-
Single dwelling	39	59.0	69	57.5	-	-	-	-	-
Multi-dwelling	27	40.9	51	42.5	-	-	-	-	-
**Religion**	-	-	-	-	0.62	0.27–1.36	0.229	-	-
Christianity	10	15.2	27	22.5	-	-	-	-	-
Islam	56	84.8	93	77.5	-	-	-	-	-
**Open defecation**	-	-	-	-	1.18	0.56–2.43	0.662	-	-
Yes	15	22.7	24	20.0	-	-	-	-	-
No	51	77.3	96	80.0	-	-	-	-	-
**Treats drinking water**	-	-	-	-	0.86	0.36–2.06	0.743	-	-
Yes	9	13.6	19	15.8	-	-	-	-	-
No	46	69.7	84	70.0	-	-	-	-	-
**Knowledge of cholera**	-	-	-	-	0.3	0.12–0.74	7.38	-	-
Yes	52	78.8	111	92.5	-	-	-	-	-
No	14	21.2	9	7.5	-	-	-	-	-
**Ate street noodles**	-	-	-	-	7.00†	1.41–34.7†	0.006†	8.74†	1.75–43.69†
Yes	7	10.6	2	1.7	-	-	-	-	-
No	59	89.4	118	98.3	-	-	-	-	-
**Ate vegetables**	-	-	-	-	1.01	0.45–2.21	0.982	-	-
Yes	21	31.8	37	30.8	-	-	-	-	-
No	18	27.3	32	48.5	-	-	-	-	-
**Ate cold fried fish**	-	-	-	-	3.90	0.94–16.14	0.045	-	-
Yes	6	9.1	3	2.5	-	-	-	-	-
No	60	90.9	117	97.5	-	-	-	-	-
**Drinking water stored in a wide-mouthed container**	-	-	-	-	0.84	0.40–1.77	0.656	-	-
Yes	13	19.7	27	22.5	-	-	-		
No	53	80.3	93	77.5	-	-	-		
**Wash your hands before eating**	-	-	-	-	1.01	0.56–1.86	0.952	-	-
Yes	30	45.5	54	45.0	-	-	-	-	-
No	36	30.0	66	55.0	-	-	-	-	-
**Wash your hands after using the toilet**	-	-	-	-	1.19	0.65–2.17	0.572	-	-
Yes	32	48.5	53	44.2	-	-	-	-	-
No	34	51.5	67	55.8	-	-	-	-	-

† , Statistically significant values.

aOR, adjusted odds ratio; OR, odds ratio; CI, confidence interval.

## Discussion

This investigation into the 2021 cholera outbreak in Adamawa State identified a high CFR and pointed out specific, modifiable high-risk factors, particularly the consumption of street-vended food and the heightened vulnerability of young children. These findings provide crucial, local evidence to guide targeted public health interventions and strengthen the national cholera control programme.

The outbreak was severe, with an overall CFR of 3.6%, a figure that far exceeds the WHO-recommended emergency benchmark of 1%.^20^ This high CFR, which is greater than that of a previous outbreak in the state in 2018, suggests significant challenges in the public health response, likely including delays in case presentation to health facilities and suboptimal clinical case management.^21^ The prolonged duration of the outbreak and its spread across multiple LGAs, as evidenced by the propagated epidemic curve, further suggest that initial control measures may have been delayed or insufficient to break the chains of person-to-person transmission.^22,23^

Our case-control study identified the consumption of street-vended noodles as a significant risk factor for cholera infection. This is consistent with findings from other urban outbreaks where contaminated food and drinks from unregulated vendors were major vehicles of transmission.^24,25,26^ In a setting like Adamawa, with known deficiencies in water and sanitation infrastructure, food items sold by street vendors are highly susceptible to contamination through poor handling, unsafe water sources and environmental exposure.^27^ Epidemiological evidence from a study in Zambia showed that contaminated food was a major path of transmission of cholera during an outbreak.^28^ Even though this study was not designed to determine whether street vendors had the knowledge or supplies to practice sanitary food and drink preparation, this area deserves further exploration.^22^ The finding in this study also contradicts other studies that did not find an association between the consumption of food from street vendors and cholera.^21,23^

In this study, younger age groups (< 10 years) were more affected than adults, which agrees with findings from some other studies that reported a higher number of cholera cases in children.^21^ Cholera among children can have significant public health implications, including high rates of morbidity and mortality, rapid spread within communities and long-term social and economic consequences.^29^ Given the increased vulnerability of this age group, they should be prioritised for oral cholera vaccination (OCV). However, given the high operational cost of OCV administration, we recommend integrating it into the routine immunisation programme in Nigeria.^30^ The cholera outbreak in Adamawa State lingered for a prolonged period, spreading across LGAs sharing borders. This showed a delayed effective response that may have delayed curtailment of the outbreak. Delays may arise during outbreak responses from multiple factors, including surveillance capacity, verification requirements, and administrative or governance-related decision-making processes.^31^

This study has several limitations inherent to outbreak investigations conducted in real-world emergency settings. Firstly, delayed outbreak notification delayed the field investigation, resulting in the analytic component being conducted in the later phase of the outbreak. However, this also allowed the inclusion of recent incident cases, thereby improving exposure recall and outcome ascertainment.

Secondly, serological testing was not performed among controls. As cholera infection may be asymptomatic, some degree of outcome misclassification is possible. This misclassification is likely to have been non-differential and would therefore bias the effect estimate towards the null, suggesting that observed associations may be conservative.

Thirdly, controls were selected from the same neighbourhoods as cases to account for shared environmental exposures. While this approach may limit variability in some risk factors, it strengthens internal validity by reducing confounding related to water and sanitation conditions.

Finally, environmental sampling was limited, and food samples were not tested. As a result, this study relied on epidemiological rather than microbiological evidence to infer transmission pathways.

## Conclusion

Our investigation into the 2021 cholera outbreak in Adamawa State concluded that children under 10 years of age and individuals consuming street-vended noodles were at significantly higher risk of infection. These findings highlight the vulnerability of young children and underscore the critical role of unregulated food sources in cholera transmission. Public health interventions included targeted hygiene education for food vendors and the community, as well as the distribution of chlorine tablets in affected areas. To mitigate future outbreaks, we recommend that the State Ministry of Health enhance food safety regulations for street vendors and sustain educational programmes that emphasise the importance of personal, food and environmental hygiene.

## References

[1] World Health Organization (WHO), Global Task Force on Cholera Control. Cholera outbreak: Assessing the outbreak response and improving preparedness [homepage on the Internet]. 2004 [cited 2021 Nov 20]. Available from: https://iris.who.int/server/api/core/bitstreams/cb9cb3d1-117f-451d-88de-0a83cb88975e/content

[2] Dureab F, Jahn A, Krisam J, et al. Risk factors associated with the recent cholera outbreak in Yemen: A case-control study. Epidemiol Health. 2019;41:e2019015. 10.4178/epih.e2019015 31010279 PMC6533552

[3] Qadri F, Khan AI, Faruque ASG, et al. Enterotoxigenic *Escherichia coli* and *Vibrio cholerae* diarrhea, Bangladesh, 2004. Emerg Infect Dis. 2005;11(7):1104–1107. 10.3201/eid1107.041266 16022790 PMC3371816

[4] Akyala IA, Shadrack BE, Ajumobi O, Olayinka A, Nguku P. Investigation of cholera outbreak in an Urban North Central Nigerian Community – The Akwanga experience. Public Health Res [serial online]. 2014 [cited 2026 May 19];4(1):7–12. Available from: http://journal.sapub.org/phr

[5] World Health Organization (WHO). Cholera global situation [homepage on the Internet]. 2023 [cited 2025 Jan 23]. Available from: https://www.who.int/emergencies/disease-outbreak-news/item/2023-DON437

[6] Mengel MA, Delrieu I, Heyerdahl L, Gessner BD. Cholera outbreaks in Africa. Curr Top Microbiol Immunol. 2014;379:117–144. 10.1007/82_2014_369 24827501

[7] Sanni F. WHO AFRO Africa regional situation updates on cholera surveillance working group [homepage on the Internet]. 2023 [cited 2025 Jan 23]. Available from: https://www.gtfcc.org/wp-content/uploads/2023/02/8th-meeting-of-the-gtfcc-working-group-on-surveillance-2023-felix-sanni.pdf

[8] World Health Organization (WHO). Cholera cases in Africa surging fast reach third 2022 [homepage on the Internet]. 2023 [cited 2025 Jan 23]. Available from: https://www.afro.who.int/news/new-cholera-cases-africa-surging-fast-reach-third-2022-total-month

[9] Nigeria Centre for Disease Control and Prevention. Cholera situation report 2021 [homepage on the Internet]. 2021 [cited 2026 Feb 01]. Available from: https://www.ncdc.gov.ng/themes/common/files/sitreps/715d0c9c284b1510e82ec4bcf97402cd.pdf

[10] Nigeria Centre for Disease Control and Prevention. Cholera situation report 2022 [homepage on the Internet]. 2022 [cited 2026 Feb 01]. Available from: https://www.ncdc.gov.ng/themes/common/files/sitreps/114c6e786ac78f09d484f070d72f2f0c.pdf

[11] Elimian KO, Musah A, Mezue S, et al. Descriptive epidemiology of cholera outbreak in Nigeria, January-November, 2018: Implications for the global roadmap strategy. BMC Public Health. 2019;19(1):1264. 10.1186/s12889-019-7559-6 31519163 PMC6743111

[12] Dan-Nwafor CC, Ogbonna U, Onyiah P, et al. A cholera outbreak in a rural north central Nigerian community: An unmatched case-control study. BMC Public Health. 2019;19(1):112. 10.1186/s12889-018-6299-3 30683078 PMC6347749

[13] Siddique AK, Islam Q, Akram K, Mazumder Y, Mitra A, Eusof A. Cholera epidemic and natural disasters; where is the link. Trop Geogr Med [serial online]. 1989 [cited 2026 May 19];41(4):377–382. Available from: http://www.ncbi.nlm.nih.gov/pubmed/2635456 2635456

[14] Adamawa State Planning Commission. Adamawa State [homepage on the Internet]. 2022 [cited 2026 Feb 01]. Available from: https://adspc.ad.gov.ng/adamawa-state/

[15] The Office for the Coordination of Humanitarian Affairs (OCHA). Northeast Nigeria: Flash Update #1 – Cholera outbreak and AWD cases in Borno, Adamawa and Yobe (BAY) states (As of 3 September 2021) [homepage on the Internet]. 2021 [cited 2026 Feb 01]. Available from: https://www.unocha.org/publications/report/nigeria/northeast-nigeria-flash-update-1-cholera-outbreak-and-awd-cases-borno-adamawa-and

[16] Elimian KO, Mezue S, Musah A, et al. What are the drivers of recurrent cholera transmission in Nigeria? Evidence from a scoping review. BMC Public Health. 2020;20(1):432. 10.1186/s12889-020-08521-y 32245445 PMC7118857

[17] Adamawa State. Water, Sanitation and Hygiene (WASH) sector 2023-2025 Medium-Term Sector Strategy (MTSS) [homepage on the Internet]. 2022 [cited 2026 Feb 01]. Available from: https://adspc.ad.gov.ng/wp-content/uploads/2025/08/Adamawa-State-2023-2025-WASH-Sector-MTSS-Report-Quality-Assured-and-Formatted-051022.pdf

[18] Federal Ministry of Health, Nigeria Centre for Disease Control, World Health Organization Regional Office for Africa, WHO Health Emergency Programme, Centers for Disease Control and Prevention. National technical guidelines for integrated disease surveillance and response [homepage on the Internet]. 2019 [cited 2025 Sep 12]. Available from: https://www.ncdc.gov.ng/themes/common/docs/protocols/242_1601639437.pdf

[19] Dutta B, Kumar N, Meshram K, Yadav R, Sodha S, Gupta S. Cholera outbreak associated with contaminated water sources in paddy fields, Mandla District, Madhya Pradesh, India. Indian J Public Health. 2021;65(5):46. 10.4103/ijph.IJPH_1118_20 33753592

[20] World Health Organization (WHO). Cholera fact sheet [homepage on the Internet]. 2024 [cited 2026 Jan 31]. Available from: https://www.who.int/news-room/fact-sheets/detail/cholera

[21] Adeneye AK, Musa AZ, Oyedeji KS, et al. Risk factors associated with cholera outbreak in Bauchi and Gombe States in North East Nigeria. J Public Health Epidemiol [serial online]. 2016 [cited 2022 Dec 19];8(11):286–296. Available from: http://www.academicjournals.org/JPHE

[22] Endris AA, Tadesse M, Alemu E, Musa EO, Abayneh A, Assefa Z. A case-control study to assess risk factors related to cholera outbreak in Addis Ababa, Ethiopia, July 2016. Pan Afr Med J. 2019;34:128. 10.11604/pamj.2019.34.128.17997 33708297 PMC7906546

[23] Maponga BA, Chirundu D, Gombe NT, Tshimanga M, Shambira G, Takundwa L. Risk factors for contracting watery diarrhoea in Kadoma City, Zimbabwe, 2011: A case control study [homepage on the Internet]. 2013 [cited 2022 Dec 19]. Available from: http://www.biomedcentral.com/1471-2334/13/567 10.1186/1471-2334-13-567 PMC4219403 24295488

[24] Ries AA, Vugia DJ, Beingolea L, et al. Cholera in Piura, Peru: A modern urban epidemic. J Infect Dis. 1992;166(6):1429–1433. 10.1093/infdis/166.6.1429 1431259

[25] Guthmann JP. Epidemic cholera in Latin America: Spread and routes of transmission. J Trop Med Hyg [serial online]. 1995 [cited 2022 Feb 04];98(6):419–427. Available from: http://www.ncbi.nlm.nih.gov/pubmed/8544225 8544225

[26] Moradi G, Rasouli MA, Mohammadi P, Elahi E, Barati H. A cholera outbreak in Alborz Province, Iran: A matched case-control study. Epidemiol Health. 2016;38:e2016018. 10.4178/epih.e2016018 27188308 PMC4967910

[27] Visa T, Emmanuel T, Mbodi F, Nguku P. Risk factors associated with cholera outbreak in Mubi Adamawa state – Nigeria, 2018. Int J Infect Dis. 2020;101(Suppl 1):266. 10.1016/j.ijid.2020.09.701

[28] Olu O, Babaniyi O, Songolo P, et al. Cholera epidemiology in Zambia from 2000 to 2010: Implications for improving cholera prevention and control strategies in the country. East Afr Med J [serial online]. 2013 [cited 2022 Feb 04];90(10):324–331. Available from: http://www.ncbi.nlm.nih.gov/pubmed/26862642 26862642

[29] Mohamed MG, Dabou EAA, Abdelsamad S, Elsalous SH. Cholera outbreaks: Public health implications, economic burden, and preventive strategies. AIMS Public Health. 2025;12(3):767–795. 10.3934/publichealth.2025039 41127425 PMC12538245

[30] Bwire G, Mwesawina M, Baluku Y, Kanyanda SSE, Orach CG. Cross-border cholera outbreaks in sub-Saharan Africa, the mystery behind the silent illness: What needs to be done? PLoS One. 2016;11(6):e0156674. 10.1371/journal.pone.0156674 27258124 PMC4892562

[31] Hassan A, Mustapha GU, Lawal BB, et al. Time delays in the response to the Neisseria meningitidis serogroup C outbreak in Nigeria – 2017. PLoS One. 2018;13(6):e0199257. 10.1371/journal.pone.0199257 29920549 PMC6007901

[32] Abdulazeez AA, Ameh C, Sadauki A, et al. Cholera outbreak investigation in Adamawa State, Nigeria 2021: An unmatched case control study. Poster session presented at: 11th TEPHINET Global Scientific Conference; 2022 Sep 04–09; Panama City, Panama; 2022.

